# The association of osteoporosis knowledge and beliefs with preventive behaviors in postmenopausal breast cancer survivors

**DOI:** 10.1186/s12905-021-01430-1

**Published:** 2021-08-11

**Authors:** Stacyann Bailey, Jenny Lin

**Affiliations:** Division of General Internal Medicine, Icahn School of Medicine at Mount Sinai, NY New York, USA

**Keywords:** Breast cancer, Postmenopausal, Osteoporosis knowledge, Preventive behaviors, Bone health

## Abstract

**Background:**

Postmenopausal breast cancer survivors (PBCS) are at increased risk of bone loss and fractures due to age-related decline of estrogen, and this risk is compounded by aromatase inhibitor cancer therapy. Several patient-level targetable risk factors can mitigate osteoporosis risk; however, adequate health behavior and risk perception in this population are underreported. The goal of this study was to evaluate osteoporosis knowledge and beliefs and assess their association with engagement in osteoporosis preventive behaviors among PBCS.

**Methods:**

In this cross-sectional descriptive study, early stage I–IIIA PBCS (ages 55–86 years) completed the Facts on Osteoporosis Quiz, Osteoporosis Health Beliefs Scale, and Osteoporosis Preventive Behaviors questionnaires. Participants who were non-English speaking or declined to participate were excluded. Clinical and sociodemographic information were obtained from chart review and baseline questionnaire, respectively. Fisher’s exact test, Student t-test, and Wilcoxon Mann–Whitney tests were used where appropriate to assess the association between knowledge and beliefs with engagement in osteoporosis preventive behaviors.

**Results:**

The mean participant age was 66.1 years with 20% self-reporting as non-Hispanic White, 40% non-Hispanic Black, 27% Hispanic, and 13% other. Approximately 83% of the cohort had estrogen receptor positive breast cancer and received a bone density scan within the last six years. Osteoporosis knowledge (10.5 ± 3.4), seriousness (14.9 ± 3.8), and susceptibility (14.0 ± 3.5) mean scores were low among PBCS. Most PBCS (75%) were adherent to calcium and vitamin D supplements, but only 47% reported engagement in strength-training exercises. Married/partnered, higher osteoporosis knowledge and health motivation scores were associated with strength-training exercise. After adjustment for marital status and osteoporosis knowledge, only health motivation score remained significantly associated with strength-training exercise (OR 5.56, 95% CI 1.35–22.93).

**Conclusions:**

PBCS are highly motivated to keep a healthy lifestyle despite limited osteoporosis knowledge, perceived risk, and susceptibility. However, < 50% participated in strength-training exercise. Our findings suggest that oncologic care should include osteoporosis and fracture prevention strategies, directed at encouraging cancer survivors to increase their engagement in osteoporosis preventive behaviors, particularly strength-training exercises.

## Background

Breast cancer is the most common cancer among women worldwide with increased incidence in postmenopausal women. Since early detection and treatment have caused a decline in breast cancer mortality, more women will die of other causes due to multiple competing risks [1, 2]. Bone loss occurs in postmenopausal women because of the natural reduction in estrogen levels with age. However, aromatase inhibitors (AIs), the standard treatment for the majority of postmenopausal women with estrogen receptor-positive breast cancer, increase bone loss at twice the rate that occurs physiologically and exacerbates the risk of fragility fractures [3]. Osteoporosis is highly relevant to the growing number of breast cancer survivors, the majority of whom will be in their sixth to eighth decade of life by 2040 [4]. The increased morbidity, reduced quality of life, and treatment-induced fractures also constitute a significant economic burden. Thus, the management of bone health is of clinical importance in postmenopausal breast cancer survivors (PBCS).

Clinical guidelines for the management of bone health in PBCS on AIs have been issued [5, 6]. The recommendation to mitigate bone loss includes bone mineral density (BMD) screening, weight-bearing exercises, dietary education, and calcium and vitamin D supplementation [7, 8], but adherence to these guidelines is inconsistent [9–11]. Recent attention has been directed towards the assessment of osteoporosis knowledge and participation in osteoporosis preventive behaviors among PBCS in efforts to increase adherence. Osteoporosis knowledge was found to be lower in PBCS compared to postmenopausal women without cancer [12]. PBCS were also more likely to be overweight or obese and consumed less fruits, vegetables, and protein than the recommended guidelines [13]. Lastly, there is a high prevalence of vitamin D deficiency and low calcium intake in this population [14].

In addition to bone loss, PBCS are more likely to develop comorbid conditions such as diabetes mellitus and cardiovascular disease [15], which are also associated with increased fracture risk [16, 17]. Consequently, PBCS may be more focused on the impact of breast cancer and managing other chronic diseases and less concerned about osteoporosis. Given that osteoporosis is asymptomatic until fracture occurs, monitoring risk factors in PBCS can help to lower the incidence and burden of the disease. In order to raise awareness of AI-induced osteoporosis, examining the level of knowledge in PBCS is one of the prerequisites that together with self-efficacy can lead to successful prevention strategies. Furthermore, PBCS should be aware that osteoporosis is serious, and there is increased susceptibility to osteoporosis because of their existing medical conditions [18]. Thus, the benefits of participating in an osteoporosis preventive strategy outweighs the perceived barriers [19].

The goal of this cross-sectional study was to determine the interrelationships between osteoporosis knowledge, health beliefs, and preventive behaviors in early stage PBCS with diabetes mellitus, which to our knowledge have not been previously reported. Using validated survey instruments, we first assessed the level of knowledge, seriousness, susceptibility, and health motivation beliefs. To capture engagement in behaviors that promote healthy bones, PBCS were also requested to disclose intake of foods that are rich in calcium and vitamin D as well as frequency of physical activity. This information will aid in the development and implementation of educational programs that increase bone density screening, self-efficacy, and disease self-management.

## Methods

### Study design

**Fig. 1:**
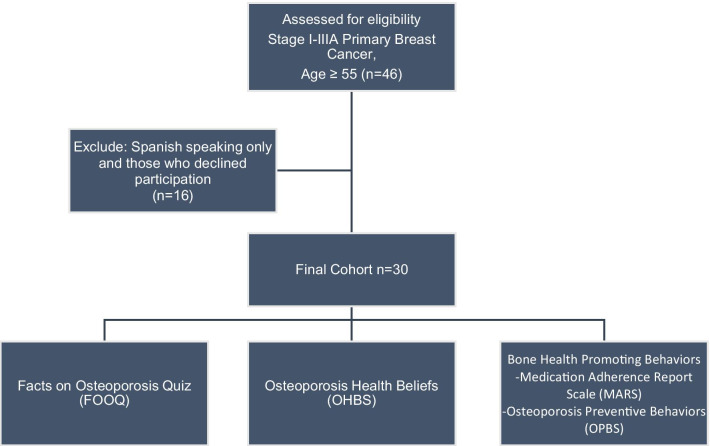
Summary of study design. The final cohort consisted of thirty early stage postmenopausal (age ≥ 55) breast cancer survivors. All participants completed four survey instruments: facts on osteoporosis quiz, osteoporosis health beliefs, medication adherence report scale and osteoporosis preventive behaviors

Participants were recruited from an ongoing longitudinal study that investigates the relationship between breast cancer and diabetes beliefs among older breast cancer survivors. The institutional review board at Mount Sinai Hospital approved the current cross-sectional study. Thirty-six patients were contacted by phone and thirty patients provided verbal informed consent. The convenience sample of thirty participants consisted of women 55 years and older with pre-existing diabetes mellitus and histologically confirmed early-stage primary breast cancer (stage I–IIIA) diagnosed within the last 5 years and without recurrence. Clinical and socio-demographic information, including receipt of bone density testing, most recent bone density scores, height, weight, body mass index, and vitamin D/calcium supplements were obtained from survey questionnaire and hospital chart review. Trained research assistants administered survey questionnaires by telephone (conducted from April 2020 to May 2020) to assess osteoporosis knowledge, beliefs, and behaviors. Patients were excluded from the study if they were non-English speaking. The study outline is summarized in Fig. 1.

### Osteoporosis knowledge

We used the Facts on Osteoporosis Quiz (FOOQ) to measure osteoporosis knowledge among the participants. FOOQ is a 20-item True–False survey instrument with a reported 0.87 content validity index [20]. The total raw score for the FOOQ was calculated as the sum of correct answers and ranged from 0 to 20. We characterized participants as having osteoporosis knowledge if they scored ≥ 14 (i.e., if they answered ≥ 70% of the items correctly).

### Osteoporosis health beliefs

We administered the susceptibility (perceived risk of developing osteoporosis), seriousness (perception of threat from having osteoporosis), and health motivation (general tendency to engage in health behaviors) subscales of the Osteoporosis Health Beliefs (OHBS) questionnaire [21]. The three subscales have been previously validated for internal consistency with Cronbach’s alpha ranging from 0.60 to 0.8 [22]. Each subscale has five questions and the response to each question carries a score from 1 (strongly disagree) to 5 (strongly agree). By summing the scores for each subscale, the total possible score for each subscale ranged from 5 to 25.

### Bone health promoting behaviors

Self-reported adherence to calcium or vitamin D supplements was assessed using the Medication Adherence Report Scale (MARS), [23] a 10-item validated adherence measure that is rated on a 5-point Likert scale (1 = “always” to 5 = “never”). Self-reported adherence was the average of the 10 questions and a score > 4.5 was defined as adherent [24]. Based on current literature, we also constructed a 7-item questionnaire to capture engagement in osteoporosis preventive behaviors such as dietary intake of calcium-containing foods and weight-bearing exercise. Participants reported the amount of daily or weekly servings for each food item (for e.g. eggs, milk, cheese, fish, green leafy vegetables, and caffeine) and the number of weekly 30-min strength-training exercise (for e.g. aerobics, jogging, dancing, and weightlifting). Participants who responded that they had ≥ 1 eight oz. calcium fortified beverage (for e.g. milk, milk substitute, or orange juice) or ≥ 1 oz. servings of cheese per day were considered engaging in osteoporosis preventive behaviors. Similarly, participants who performed at least one 30-min session of weight-bearing exercises weekly were considered adherent to exercise.

### Statistical analysis

We report descriptive statistics for the clinical and sociodemographic characteristics of the study participants. Fisher’s exact test was used to determine the association between dichotomized variables osteoporosis knowledge (1 = knowledge score ≥ 14, 0 = knowledge score < 14) and receipt of bone density scan with osteoporosis preventive behaviors (diet/exercise, adherence to calcium/vitamin D supplements). Student t-test and Wilcoxon Mann–Whitney tests were used to assess the association between continuous knowledge and beliefs scores with engagement in osteoporosis preventive behaviors. Multivariable logistic regression assessed for the association between osteoporosis preventive behaviors (diet/exercise) and beliefs (health motivation) after adjusting for knowledge and marital status. All analyses were conducted using SAS Statistical software version 9.4 (SAS Institute Inc., Cary, NC).

## Results

### Patient characteristics

Thirty PBCS completed the questionnaires, and their characteristics are summarized in Table 1. The mean participant age was 66.1 years (standard deviation [SD] = 7.91). Our cohort was racially and ethnically diverse with 20% self-reporting as non-Hispanic White, 40% non-Hispanic Black, 27% Hispanic, and 13% other. The majority of participants (63%) had more than a high school education, earned more than $1500 per month (53%), and were not married or partnered (77%). The mean height and median weight were 161.7 cm (SD = 7.2) and 76.95 kg (interquartile range [IQR]: 71–90), respectively, and 57% of this cohort was obese (Body Mass Index [BMI] ≥ 30). The distribution of breast cancer stages was: 10.3% stage 0, 41.4% stage I, 37.9% stage II, and 10.3% stage III, and 83% were estrogen receptor positive, 70% were progesterone receptor positive, and 17% were human epidermal growth factor receptor 2 positive. The majority (90%) were receiving hormone therapy in the form of aromatase inhibitor. Out of the 30 participants, 25 had received at least one bone density scan by dual-energy x-ray absorptiometry (DEXA) within the last six years. The T-scores for those who had received a DEXA were: 0.28 ± 1.13 at the lumbar spine, − 0.70 (− 1.70–0) at the femoral neck, and − 0.04 ± 1.15 for the total hip.

**Table 1:** Clinical and socio-demographic characteristics of postmenopausal breast cancer survivor

Characteristics	
Mean age (years), ± SD	66.1 ± 7.91
Race/Ethnicity, N (%)	
White	6 (20)
Black	12 (40)
Latino/Hispanic	8 (27)
Other	4 (13)
High school education or more, N (%)	19 (63)
Monthly income > *$1500,* N (%)	16 (53)
Married, N (%)	7 (23)
Mean height (cm), ± SD	161.7 ± 7.2
Median weight (kg), IQR	76.95 (71–90)
Mean Body Mass Index (kg/cm^2^), ± SD	31.2 ± 5.8
Breast cancer stage, N (%)	
Stage 0	3 (10.3)
Stage I	12 (41.4)
Stage II	11 (37.9)
Stage III	3 (10.3)
Aromatase inhibitor therapy, N (%)	27 (90)
Receipt of bone density screening (DEXA), N (%)	25 (83)
T-score	
Mean lumbar spine (± SD)	0.28 ± 1.13
Median femoral neck (IQR)	− 0.70 (− 1.70–0)
Mean total hip (± SD)	− 0.04 ± 1.15
Mean osteoporosis knowledge score, ± SD	10.5 ± 3.4
Osteoporosis health beliefs	
Mean susceptibility score (± SD)	14.0 ± 3.5
Mean seriousness score (± SD)	14.9 ± 3.8
Median health motivation score (IQR)	19 (18–20)
Osteoporosis preventive behaviors, N (%)	
Calcium fortified beverage	14 (47)
Caffeinated beverage	18 (60)
Cheese	14 (47)
Fish	11 (37)
Eggs	5 (17)
Strength-training exercises	14 (47)
Calcium/Vitamin D supplement adherence	15 (75)

### Osteoporosis knowledge and beliefs

The mean score on the FOOQ was 10.5 (SD = 3.4). The majority of participants (83%) correctly answered questions regarding whether “osteoporosis affects men and women,” that “there are many ways to prevent osteoporosis,” and that “there are treatments for osteoporosis after it develops.” However, 93% of participants answered incorrectly that “walking has a great effect on bone health.” Overall, only 23% of participants were classified as having osteoporosis knowledge (≥ 70% correct). Regarding health beliefs, the susceptibility to osteoporosis subscale had the lowest mean score (14.0 ± 3.5), although the majority (53%) agreed or strongly agreed that their “chances of getting osteoporosis are high.” Similarly, on the seriousness subscale, the mean score was (14.9 ± 3.8), and most participants (73%) agreed “it would be very serious if [I] got osteoporosis.” Interestingly, participants had the highest scores on the health motivation subscale (median: 19 [IQR: 18–20]), and 80% agreed that they followed recommendations to keep healthy.

### Osteoporosis health behaviors

Twenty (67%) participants reported that they were actively taking calcium or vitamin D supplements on the MARS questionnaire and their responses were corroborated with chart review. The mean medication adherence score was 4.73 (SD = 0.45), indicating that most participants were adherent to these supplements. Only 47% of participants reported a daily intake of at least one 8 oz. calcium-fortified beverage; similarly, only 47% reported consuming at least one 1 oz. serving of cheese daily. In addition, 47% of participants reported that they engaged in one or more 30-min session of strength-training exercises weekly.

### Characteristics associated with engagement in osteoporosis preventive behaviors

In non-parametric analysis, participants who received a bone density scan had a significantly higher calcium/vitamin D supplement adherence score compared to those who did not receive a bone density scan (median score 5.0 [IQR: 4.9–5.0] vs. 3.9 [IQR: 3.7–4.1], *p* = 0.02). When medication adherence was dichotomized (1 = mean adherence score ≥ 4.5; 0 = mean adherence score < 4.5), 83% of those who received a bone density scan were adherent to calcium/vitamin D supplements compared to 0% of those who had not received a bone density scan (*p* = 0.05). There was also a significant association between receipt of bone density scan and calcium beverage intake (56% vs. 0%, *p* = 0.04). We observed no association between receipt of bone density scan and osteoporosis knowledge, health beliefs, or diet/exercise.

The proportion of women who identified as black (17%) and consumed ≥ 1 oz. serving of cheese daily was lower than the proportion of women who identified as Hispanic (37%), white (100%), and other (75%) [*p* = 0.003]. Additionally, married women and those with more than a high school education were more likely to report eating ≥ 1 oz. serving of cheese daily (86% vs. 35% [*p* = 0.03] and 63% vs. 18% [*p* = 0.03], respectively). Those who were married were also more likely to participate in one or more 30-min strength-training exercises weekly (86% vs. 35% [*p* = 0.03], Table 2). We observed a significant difference in osteoporosis knowledge scores between those who engaged in ≥ 1 30-min strength training exercise weekly and those who did not (mean knowledge score 12.3 vs. 8.9, *p* = 0.004). When osteoporosis knowledge was dichotomized, 86% of knowledgeable participants engaged in strength training exercises compared to 35% low knowledgeable participants (*p* = 0.03). Similarly, those who engaged in strength training exercises had higher health motivation scores (median score 20 [IQR: 20–21] vs. 18 [IQR: 17–18.5], *p* = 0.0002), Table 2). In a multivariable logistic regression model, health motivation scores remained significantly associated with exercise (OR 5.56, 95% CI 1.35–22.93), after adjusting for marital status and osteoporosis knowledge. We observed no association between osteoporosis knowledge or health beliefs and adherence to calcium/vitamin D supplements.

**Table 2:** Patient characteristics associated with adherence to strength-training exercises

Characteristics	Adherent	Non-adherent	*p*-value
Mean age (years), ± SD	66.4 ± 8.45	65.9 ± 7.67	0.9
Race/Ethnicity, N (%)			0.09
White	5 (83)	1 (17)	
Black	5 (42)	7 (58)	
Latino/Hispanic	4 (50)	4 (50)	
Other	0 (0)	4 (100)	
Married, N (%)	6 (86)	1 (14)	**0.03**
Monthly income > $1500, N (%)	9 (56)	7 (44)	0.3
High school education or more, N (%)	10 (53)	9 (47)	0.5
Mean Body Mass Index (kg/cm^2^), ± SD	30.7 ± 3.56	31.7 ± 7.32	0.7
Receipt of bone density screening (DEXA), N (%)	12 (48)	13 (52)	1.0
T-score			
Mean lumbar spine (± SD)	0.33 ± 1.03	0.23 ± 1.28	0.8
Median femoral neck (IQR)	− 1.0 (− 1.55–0.55)	− 0.60 (− 1.8–0.0)	0.6
Mean total hip (± SD)	0.04 ± 1.15	− 0.11 ± 1.20	0.8
Mean osteoporosis knowledge score, ± SD	12.3 ± 2.84	8.94 ± 3.04	**0.004**
Osteoporosis health beliefs			
Mean susceptibility score (± SD)	13.9 ± 4.6	14.13 ± 2.25	0.8
Mean seriousness score (± SD)	14.2 ± 4.28	15.5 ± 3.41	0.4
Median health motivation score (IQR)	20 (20–21)	18 (17–19)	**0.0002**
Calcium/Vitamin D supplement adherence, N (%)	6 (40)	9 (60)	1.0

The bold values represent a signification association between the characteristics and strength training exercises

## Discussion

Bone loss is associated with aging and long-term aromatase inhibitor therapy for women with breast cancer. As such, postmenopausal breast cancer survivors (PBCS) are at a greater risk for osteoporosis and fragility fractures compared to other postmenopausal women. While osteoporosis is incurable, it is widely recognized as a treatable and preventable disease. In PBCS, several guidelines have supported the use of bone modifying agents to prevent bone loss and fractures. However, initiation and maintenance of healthy bone behaviors play an essential role in delaying or preventing pharmacological intervention and is the most cost-effective approach. Moreover, the factors related to an individual’s engagement in healthy bone behaviors are complex and have not been reported in PBCS. In this pilot study, we provide the first assessment of osteoporosis knowledge, health beliefs, and engagement in healthy bone behaviors and explored the interrelationships between these three domains in PBCS. We found that the majority of PBCS (90%) were receiving adjuvant hormonal therapy in the form of aromatase inhibitor and had received a bone density scan (83%) within the last six years. However, osteoporosis knowledge was low in this population, with an average correct score of 55%. Participants did not perceive that they were susceptible to osteoporosis and did not perform weight-training exercises regularly. Although most participants were adherent to calcium/vitamin D supplements, they had very low dietary calcium intake.

We found several sociodemographic characteristics that were related to low osteoporosis knowledge among our study participants. PBCS who were not married, had less than a high school education, or lower monthly income were less knowledgeable about osteoporosis. These results are consistent with previously reported studies in postmenopausal women [25–27] and PBCS [12, 28]. Interestingly, 80% of our participants knew that bone loss normally speeds up after menopause. However, we did not observe a significant association between bone density score at any site (proximal femur, total femur, or lumbar spine) or receipt of bone density scan with osteoporosis knowledge. Although the lack of association between bone density screening and osteoporosis knowledge was also demonstrated in previous studies [29, 30], it is important to note that these studies were not conducted in PBCS and with competing demands from multiple illnesses such as diabetes, all of which greatly increase risk for bone fractures. This suggests that the personal experience of receiving a bone density scan does not improve osteoporosis knowledge. We also found that many participants (57%) recognized that a lifetime of low calcium/vitamin D intake increases the risk of osteoporosis, but this knowledge did not translate to adherence to calcium/vitamin D supplements. Participants with poor osteoporosis knowledge may be more reliant on calcium/vitamin D supplements and not as aware of the importance of also including calcium rich foods in their diet or knowing which foods are high in calcium. Thus, health care providers should better educate older breast cancer survivors who are at higher risk of developing osteoporosis about osteoporosis risk factors and preventive behaviors.

Our study participants had low sense of seriousness and perceived risk of osteoporosis. This is consistent with participants without breast cancer but of similar age, menopausal status, and bone density scores reported in other studies [31, 32]. It is plausible that PBCS believe osteoporosis is not a serious threat and have low perceived risk of developing osteoporosis if they do not have any clinical evidence or symptoms of osteoporosis such as low bone density scores, history of fracture, or bone pain. Hsieh et al. [33] showed that compared to breast cancer, cardiovascular disease, and neurological disorders, most women were less concerned about osteoporosis and therefore had lower perceived susceptibility. Given that our population were all PBCS, they may have perceived osteoporosis to be much less serious or risky compared to breast cancer. Additionally, more than half our cohort was obese and overall, there were few participants who had a diagnosis of osteopenia, so it is possible that they may have felt less susceptible. In fact, we did find that those with lower lumbar bone density scores had higher susceptibility scores. This suggests that knowledge of their bone density score may increase perceived susceptibility to osteoporosis.

Osteoporosis knowledge and health beliefs were significantly associated with engagement in osteoporosis preventive behaviors. In particular, those with higher knowledge and health motivation scores were more likely to engage in strength-training exercises. We also found that marital status was associated with exercise, but only health motivation remained significantly associated with exercise after adjustment for all three characteristics. While walking is beneficial in promoting overall fitness and improvement in quality of life, weight-bearing (aerobics, jogging, dancing) and resistance exercises (weight-training) increase bone mineral density in older patients with osteoporosis [34]. PBCS, several studies have also shown that weight-bearing exercises exert a positive effect on bone metabolism by increasing bone formation markers and lean muscle mass while preserving bone mineral density, all of which are important factors in reducing falls and consequently risk of bone fractures [35, 36]. Thus, finding ways to increase health motivation among older breast cancer survivors may help increase their engagement in strength training exercises which can not only decrease their risk for developing osteoporosis but may also improve their breast cancer prognosis.

The results of the current study are limited due to a modest sample size, which enabled us to detect weaker associations between osteoporosis knowledge, health beliefs and behaviors. Adherence to supplements, exercise, and diet are not specific to osteoporosis and may reflect a general tendency to keep a healthy lifestyle to prevent or treat other conditions. The study is also cross-sectional in design conducted at a single medical institution, thus limiting generalizability. However, our cohort was diverse in terms of race and socioeconomic status making it representative of the community and other urban areas in the United States. Finally, the majority of our participants had a prior bone density scan and were on AI therapy which may have affected their perceptions and behavior to osteoporosis.

The results of the present study have implications for practice, future research, and policy. First, given the low knowledge and perceived risk among PBCS, it is possible that they are more concerned with managing their breast cancer and diabetes diagnoses and less concerned about osteoporosis. Future research should explore the interrelationship between breast cancer, diabetes, osteoporosis beliefs, and their impact on behaviors that promote bone health. Second, clinical guidelines for bone health in breast cancer patients on AIs have been issued but compliance within the oncological community is inconsistent. Barriers that prevent translating these guidelines into practice should be identified to reduce future suboptimal or inadequate treatment of bone health. Lastly, participation in strength-training exercises and adherence to dietary calcium intake were low among participants, and greater health motivation was the only factor associated with exercise. This finding supports implementation of osteoporosis preventive programs in breast cancer care directed towards promoting health motivation and increasing strength-training exercise.

## Conclusions

In this study, we assessed the association between osteoporosis knowledge, beliefs, and engagement in osteoporosis preventive behaviors among PBCS, a unique population at increased risk for bone loss and fractures. Despite low knowledge, perceived risk, and seriousness of osteoporosis, PBCS believed in the importance of keeping healthy and followed recommendations such as adherence to calcium/vitamin D supplement intake. However, similar to the general population, the majority did not engage in sufficient strength-training exercises. Our results suggest that educating PBCS about osteoporosis and the seriousness and risk of developing osteoporosis may help encourage them to engage in osteoporosis-preventive behaviors to mitigate bone deterioration.

## Data Availability

The data that support the findings of this study are available from the Surveillance, Epidemiology and End Results (SEER)-Medicare Database of the National Cancer Institute (NCI) but restrictions apply to the availability of these data, which were used under license for the current study, and so are not publicly available. Data are however available from the authors upon reasonable request and with permission of NCI.

## Abbreviations

PBCS: Postmenopausal Breast Cancer Survivors
AI: Aromatase Inhibitor
BMD: Bone Mineral Density
FOOQ: Facts on Osteoporosis Quiz
DEXA: Dual-Energy X-ray Absorptiometry
OHBS: Osteoporosis Health Beliefs
MARS: Medication Adherence Report Scale
